# The Secretome of Human Trophoblast Stem Cells Attenuates Senescence‐Associated Traits

**DOI:** 10.1111/acel.70368

**Published:** 2026-01-11

**Authors:** Kotb Abdelmohsen, Jennifer L. Martindale, Martina Rossi, Chang Hoon Shin, Apala Pal, Rachel Munk, Martin Salamini‐Montemurri, Mirko Baranzini, Ethan M. Arends, Maja Mustapic, Yuta Lee, Jau‐Nan Lee, Sicco H. Popma, Justin Hu, Nathan Duda, Carlos J. Nogueras‐Ortiz, Dimitrios Kapogiannis, Chang‐Yi Cui, Myriam Gorospe

**Affiliations:** ^1^ Laboratory of Genetics and Genomics National Institute on Aging (NIA) Intramural Research Program (IRP), National Institutes of Health (NIH) Baltimore Maryland USA; ^2^ Translational Gerontology Branch National Institute on Aging (NIA) Intramural Research Program (IRP), National Institutes of Health (NIH) Baltimore Maryland USA; ^3^ Laboratory of Clinical Investigation National Institute on Aging (NIA) Intramural Research Program (IRP), National Institutes of Health (NIH) Baltimore Maryland USA; ^4^ Accelerated Biosciences Corp Philadelphia Pennsylvania USA

**Keywords:** aging, cellular senescence, extracellular vesicles (EVs), human trophoblast stem cells, inflammation, SASP, secretome, senotherapeutics

## Abstract

Senescent cells display indefinite growth arrest and a pro‐inflammatory, senescence‐associated secretory phenotype (SASP). As the accumulation of senescent cells in tissues with age plays detrimental roles in age‐related pathologies, there is much interest in finding therapeutic strategies to eliminate them or suppress the SASP. In this study, we investigated the impact of the secretome and extracellular vesicles (EVs) derived from human trophoblast stem cells (hTSCs) on senescent human fibroblasts. We found that the hTSC conditioned medium (hTSC‐CM), and in particular the EVs (hTSC‐EVs), significantly reduced the levels of mRNAs encoding SASP factors and the secretion of SASP factors including CXCL1, IL8, and GDF15. Proteomic analysis of hTSC‐CM and EVs indicated an enrichment in proteins involved in cell adhesion, tissue repair, and remodeling of the extracellular matrix (ECM). Furthermore, incubation of senescent cells with hTSC‐EVs attenuated DNA damage and inflammatory signaling, at least in part by suppressing the function of NF‐κB, a major transcriptional regulator of the SASP program. Our findings underscore the value of hTSC‐CM and EVs therein in therapeutic approaches directed at senescent cells.

## Graphical abstract

Human trophoblast stem cell‐derived secretome/conditioned medium (hTSC‐CM) and the extracellular vesicles (EVs) therein suppress DNA damage and NF‐κB activation in senescent fibroblasts, in turn reducing the production of senescence‐associated secretory phenotype (SASP) factors. This study highlights hTSC‐CM and EVs as potential senotherapeutic agents.

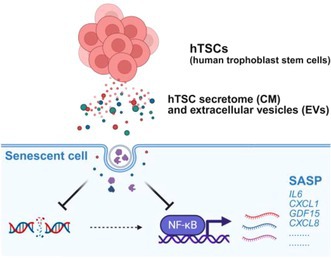

## 1 Introduction

Cellular senescence is a state of persistent cell cycle arrest in response to various stressors, including DNA damage, oxidative injury, and telomere shortening (Blagosklonny 2023; Kumari and Jat 2021). Key features of senescent cells include aberrant organelle function, morphological changes, and the acquisition of a senescence‐associated secretory phenotype (SASP). The SASP is largely orchestrated by signaling pathways that activate the transcription factor NF‐κB, in turn initiating a program to synthesize pro‐inflammatory molecules, matrix‐remodeling metalloproteases, and growth factors (Ohtani 2022; Zhao et al. 2024). While cell senescence and the SASP contribute beneficially to tissue repair, immune surveillance, and tumor suppression at younger ages, they contribute detrimentally to aging and age‐related diseases later in life (Ohtani 2022; Zhao et al. 2024). In this regard, the accumulation of senescent cells in aged tissues can aggravate pathologies including neurodegeneration, cancer, and cardiovascular diseases (Shreeya et al. 2024; Baar et al. 2017; Kovacic et al. 2011; Wang et al. 2024). As the SASP is the main damaging component of the senescent cell secretome (Basisty et al. 2020), there is heightened interest in developing therapeutic strategies aimed at selectively eliminating senescent cells and/or modulating the SASP to alleviate its harmful effects (Roger et al. 2021; Rossi and Abdelmohsen 2021; Zhao et al. 2024).

Extracellular vesicles (EVs) are small particles enclosed by a lipid bilayer and are secreted by most cell types. EVs contain DNA, RNA, lipids, and proteins, and can mediate cell‐to‐cell communication and influence gene expression patterns in recipient cells (Kumar et al. 2024). Recent studies have highlighted the critical roles of EVs as key mediators of the SASP, with increased production and altered cargoes contributing to both the beneficial and detrimental effects of the SASP (Wallis et al. 2020). For example, EVs secreted from senescent cells promote paracrine senescence in neighboring cells by delivering interferon‐induced transmembrane protein 3 (IFITM3) (Borghesan et al. 2019; Estévez‐Souto et al. 2023). Additionally, small extracellular vesicles (sEVs) secreted by senescent cells, enriched in reactive oxygen species (ROS)‐induced EphA2, can promote cancer cell proliferation (Takasugi et al. 2017).

However, the detrimental effects of the SASP can be counteracted by opposing secretomes from other cells. For example, there is growing evidence that the beneficial effects of stem cells, such as the suppression of senescence‐associated inflammation and the promotion of tissue repair, are largely mediated through their secretome, which includes both soluble factors and EVs (Praveen Kumar et al. 2019). In addition, heterochronic parabiosis studies showed that exposure to a young systemic environment rejuvenates old tissues by restoring regenerative signaling through circulating factors (EVs and non‐EV particles) without the need for stem cell engraftment (Conboy et al. 2005). Mesenchymal stem cell‐derived extracellular vesicles (MSC‐EVs) can suppress inflammation, accelerate muscle regeneration, and enhance proliferation, providing protection against cellular senescence in mouse models of muscle injury (Sandonà et al. 2021). MSC‐EVs reverse senescent cell phenotypes by lowering the production of senescence proteins p16, p21, and p53, by restoring the balance between pro‐ and anti‐inflammatory cytokines, and by reducing oxidative damage and counteracting age‐associated mitochondrial dysfunction (Gorgun et al. 2022). The anti‐senescence properties of MSC‐EVs appear to be elicited through the transfer of specific microRNAs or proteins that modulate senescence‐associated pathways (Xiao et al. 2022; Alfonzo et al. 2022; Motlagh et al. 2024). These findings highlight the potential value of MSC‐EVs in the development of senotherapeutic strategies aimed at mitigating the effects of aging and age‐related diseases (Dorronsoro et al. 2021). Unfortunately, the wide use of MSC‐EVs is hindered by several limitations, particularly poor immune tolerance towards MSC‐EVs and the difficulty in scaling up their production.

In contrast, human trophoblast stem cells (hTSCs) can be produced at scale, as they divide rapidly. Additionally, TSCs exhibit immune privilege due to the expression of HLA‐G, which has important therapeutic value (Contini et al. 2020). However, the secretome of hTSCs and associated EVs has not been studied in the context of senescence. Here, we investigated the impact of the hTSC secretome (conditioned medium, CM) and EVs therein on WI‐38 fibroblasts, derived from human lung. After triggering senescence in WI‐38 fibroblasts by exposure to ionizing radiation (IR) or etoposide (Eto), incubation with hTSC‐CM lowered the expression levels of SASP‐associated transcripts (*IL8*, *CXCL1*, *GDF15*, *TGFB1*, *BAFF*, and *ICAM1* mRNAs). A similar reduction was observed following incubation with hTSC‐EVs. Proteomic analysis of the senescent WI‐38 cell secretome confirmed that the corresponding proteins and other SASP factors were similarly reduced. Proteomic characterization of the hTSC‐CM and hTSC‐EVs revealed an enrichment of proteins involved in proliferation and extracellular matrix remodeling. Moreover, treatment with hTSC‐CM and hTSC‐EVs reduced IR‐induced DNA damage and phosphorylation of NF‐κB, a key regulator of the SASP program. These results highlight the potential of hTSC‐CM and EVs as therapeutic agents to mitigate senescence and its associated effects, underscoring their potential value for combating age‐associated diseases.

## 2 Results

### 2.1 hTSC‐CM Suppresses SASP mRNAs

**FIGURE 1:**
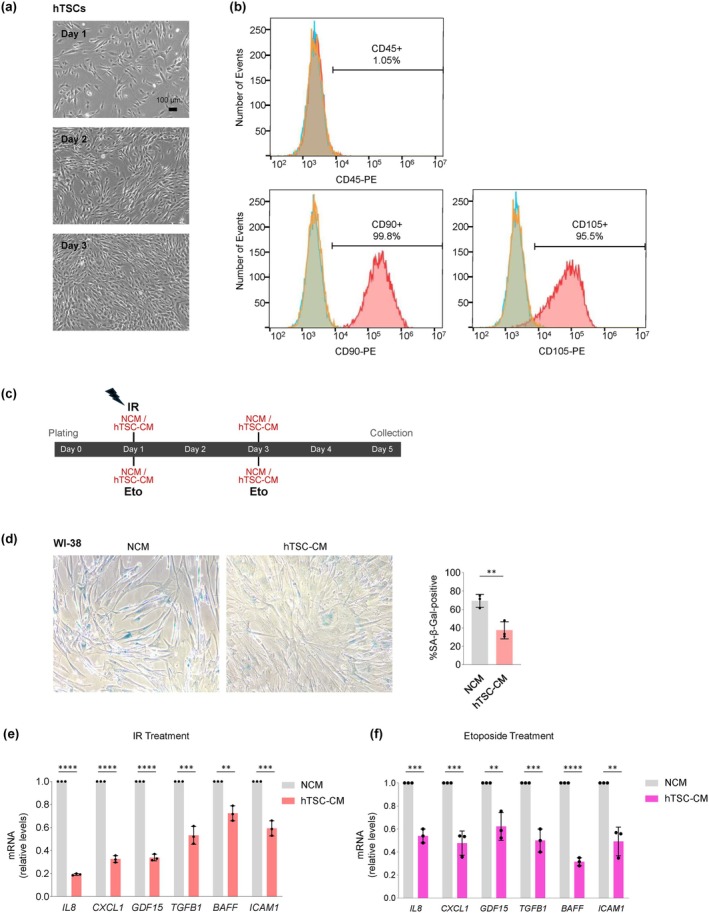
Incubation with hTSC‐CM reduces the levels of SASP mRNAs in senescent WI‐38 fibroblasts. (a) Phase‐contrast micrographs of hTSCs (passage 7) on three consecutive days of culture (Days 1–3). (b) Flow cytometry analysis of surface markers of hTSCs at passage 7 using antibodies recognizing stem cell markers CD90 and CD105, and the pan‐hematopoietic marker CD45 (to identify possible contamination). (c) Experimental timeline and workflow for inducing senescence in WI‐38 human lung fibroblasts by a single exposure to ionizing radiation (IR, 10 Gy) or by continuous treatment with etoposide (Eto), incubated in the presence of non‐conditioned medium (NCM) or conditioned medium from hTSC (hTSC‐CM) from Day 1 to Day 5; on Day 3, NCM or hTSC‐CM was refreshed with nothing additional (IR group) or with fresh Eto (Eto group). On Day 5, cells were harvested for analysis. (d) Senescence‐associated β‐galactosidase (SA‐β‐Gal) activity in WI‐38 cells on Day 5 after IR, comparing the effect of culture in hTSC‐CM or NCM as visualized on micrographs (*left*) and quantified (*right*) to represent % SA‐β‐Gal‐positive WI‐38 cells relative to total cells per field. (e, f) WI‐38 cells were exposed to IR (e) or Eto (f) and then incubated in either NCM or hTSC‐CM for 5 days, whereupon the levels of SASP‐related mRNAs were measured by RT‐qPCR analysis and normalized to the levels of *GAPDH* mRNA. Data in (d‐f) represent the means ±SEM. Statistical significance (*p < 0.05; **p < 0.01; ***p < 0.001; ****p < 0.0001) was assessed using Student's *t*‐test.

Passage 7 hTSCs grew robustly in culture, as assessed on phase‐contrast micrographs (Figure 1a), and were a highly pure population, displaying canonical stem cell markers CD90 and CD105 (> 95% positive) and minimal levels of the pan‐hematopoietic marker CD45 (1.05% positive), as assessed by flow cytometry (Figure 1a). These features persisted through passages 11 and 19 (Figure S1a). To evaluate the impact of hTSC‐CM on senescent cells, we prepared conditioned medium (CM) collected from hTSCs (Methods) and used fresh, non‐conditioned medium (NCM) in control incubations. We triggered senescence employing a well‐established cell model, human diploid WI‐38 fibroblasts, using two different paradigms. In one, WI‐38 fibroblasts were exposed to ionizing radiation (IR, 10 Gy) once on Day 1 after plating (Day 0); IR causes single‐ and double‐strand breaks on the DNA that promote senescence and activate NF‐κB, which in turn increases the transcription of genes encoding SASP factors. Immediately after exposure to IR, cells were incubated for 5 days with either hTSC‐CM or NCM, refreshing the medium on Day 3, as shown (Figure 1c). In the other, etoposide (Eto, 50 μM) was added to the culture medium on Day 1, and refreshed on Day 3; cells were collected on Day 5. The continuous exposure to Eto stabilizes TOP2‐DNA cleavage complexes in cells, which generates double‐strand DNA breaks that promote senescence, activate NF‐κB, and enhance the SASP trait. Along with the addition of Eto, WI‐38 fibroblasts also received NCM or hTSC‐CM on Days 1 and 3 (Figure 1c).

To evaluate the effect of hTSC‐CM relative to the control incubation (NCM) on senescence traits, we performed several measurements. By Day 5, IR‐treated fibroblasts incubated with hTSC‐CM displayed lower percentages of cells positive for senescence‐associated β‐galactosidase activity (SA‐β‐Gal) than fibroblasts incubated with NCM (Figure 1d); likewise, both the density and numbers of IR‐treated fibroblasts increased when cultured in hTSC‐CM as compared to NCM (Figure S1b). By Day 5, IR‐treated fibroblasts cultured in hTSC‐CM or NCM were collected, and reverse transcription (RT) followed by quantitative (q)PCR analysis was used to measure the levels of several mRNAs. As shown, fibroblasts cultured with hTSC‐CM expressed higher levels of *MKI67* mRNA, encoding the proliferation marker Ki‐67 (Mansfield et al. 2024), than fibroblasts cultured in NCM (Figure S2c). Conversely, the levels of SASP‐related transcripts, including *IL8*, *CXCL1*, *GDF15*, *TGFB1*, *BAFF*, and *ICAM1* mRNAs, were significantly lower in WI‐38 cells incubated with hTSC‐CM than in cells incubated with NCM (Figure 1e).

We validated the specificity of the effects of hTSC‐CM after triggering senescence in WI‐38 cells by exposure to Eto following the same timeline as for IR (Figure 1c). Similarly to what was seen after IR, incubation with hTSC‐CM markedly decreased the levels of SASP‐associated transcripts after Eto‐induced senescence, as compared with incubation with NCM (Figure 1f). Collectively, these findings indicate that in cells induced to undergo senescence, incubation with hTSC‐CM dampened senescence traits, as it reduced growth arrest, SA‐β‐Gal activity, and the expression of mRNAs encoding SASP factors.

### 2.2 Reduced Expression and Secretion of SASP Factors by Fibroblasts Treated With hTSC‐CM

**FIGURE 2:**
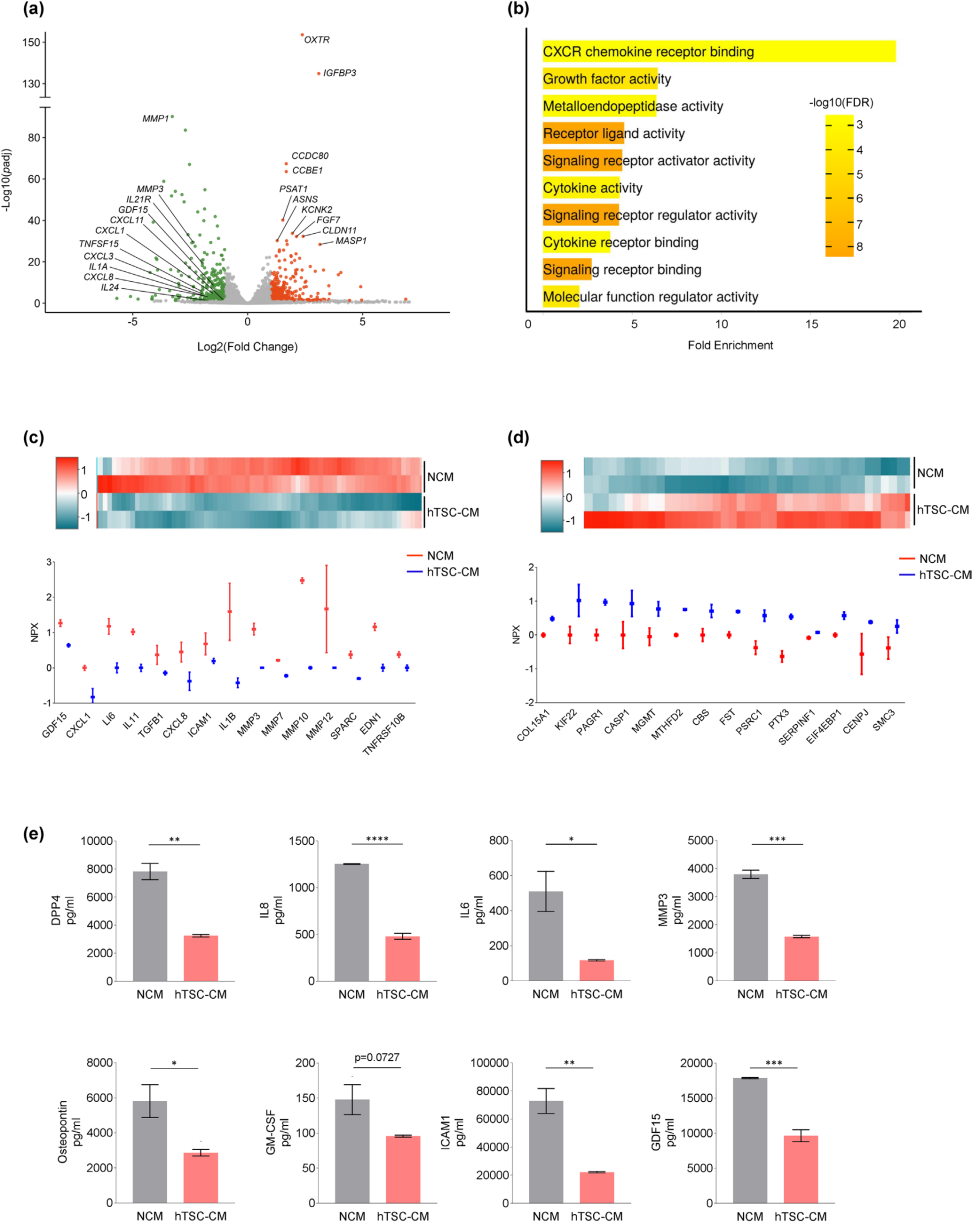
Broad suppression of production and secretion of SASP factors in IR‐treated WI‐38 fibroblasts incubated in hTSC‐CM. (a) Volcano plot summarizing RNA‐seq analysis of the relative abundance of SASP‐related mRNAs in WI‐38 cells that were exposed to IR and then cultured with either hTSC‐CM or NCM. (b) Those mRNAs from panel (a) showing reduced expression in the hTSC‐CM relative to NCM were used to identify pathways enriched by Gene Ontology (GO) using ShinyGO. (c, d) Olink normalized proteomic analysis (NPX) of proteins less abundant [shown in green (c)] or more abundant [shown in red (d)] in the secretome of IR‐treated WI‐38 cells by 5 days following culture with hTSC‐CM relative to NCM. *Top*, heatmaps of the analysis; *bottom*, subsets of the secreted factors. (e) Measurement of SASP factors (DPP4, IL8, IL6, MMP3, ICAM1, and GDF15) secreted by irradiated WI‐38 cells that were cultured in hTSC‐CM or NCM using Bio‐Plex. Data in (e) represent the means ±SEM. Statistical significance (*p < 0.05; **p < 0.01; ***p < 0.001; ****p < 0.0001) was assessed using Student's *t*‐test.

To comprehensively evaluate the effects of hTSC‐CM on senescent cells, we conducted high‐throughput analysis of the transcriptome and secretome of WI‐38 cells after exposing them to senescence‐inducing damage and culturing them in hTSC‐CM or NCM. Following exposure to IR, WI‐38 fibroblasts were incubated with hTSC‐CM or NCM; on Day 5, RNA‐sequencing (RNA‐seq) analysis (GSE282054) revealed a broad reduction in the levels of SASP‐associated mRNAs in WI‐38 fibroblasts incubated with hTSC‐CM relative to those incubated in NCM. Among the reduced mRNAs in fibroblasts incubated with hTSC‐CM were those that encode SASP factors (*CXCL1*, *IL8*, and *GDF15* mRNAs), and particularly several matrix metalloproteinases (*MMP1*, *MMP3*, and *MMP9* mRNAs), known mediators of inflammation, extracellular matrix remodeling, and tissue damage (Figure 2a and Table S1). Gene Ontology (GO) analysis of mRNAs showing reduced abundance, performed using ShinyGO, revealed that several biological pathways associated with the SASP were affected by incubation with hTSC‐CM, including the CXCR chemokine receptor binding pathway, metalloendopeptidase activity, cytokine activity, and signaling receptor ligand activity (Figure 2b). GO analysis of mRNAs showing increased abundance indicated an enrichment in pathways involved in ECM collagen, plasma membrane signaling, and structural integrity (Figure S1). These findings suggest that culture with hTSC‐CM not only suppressed SASP‐related inflammation but it also actively promoted ECM integrity, fostering a more regenerative cellular environment.

To further assess the impact of hTSC‐CM on the secretion of SASP factors, we performed Olink proteomic analysis to compare the secretory profiles of WI‐38 fibroblasts cultured in hTSC‐CM relative to those cultured in NCM (Table S2). Several pro‐inflammatory cytokines, including IL6, GDF15, and CXCL1, were less abundant in the hTSC‐CM‐treated group (Figure 2c), mirroring the RNA‐seq data, and suggesting a coordinated suppression of both mRNAs encoding SASP factors and the secretion of such SASP factors by senescent WI‐38 cells in the presence of hTSC‐CM. Several other proteins were more abundantly secreted by WI‐38 cells after incubation with hTSC‐CM (Figure 2d), suggesting a broad reprogramming of the secretory profile.

The results obtained using the Olink platform were further evaluated by Bio‐Plex analysis. In the same paradigm of fibroblasts treated with IR and incubated with hTSC‐CM or NCM, Bio‐Plex analysis confirmed a reduction in the levels of individual SASP factors (IL6, IL8, GDF15, MMP3, and others) after incubation with hTSC‐CM (Figure 2e). Collectively, these findings support the notion that hTSC‐CM broadly suppresses the production and secretion of SASP factors by senescent fibroblasts.

### 2.3 Characterization of the hTSC‐CM

**FIGURE 3:**
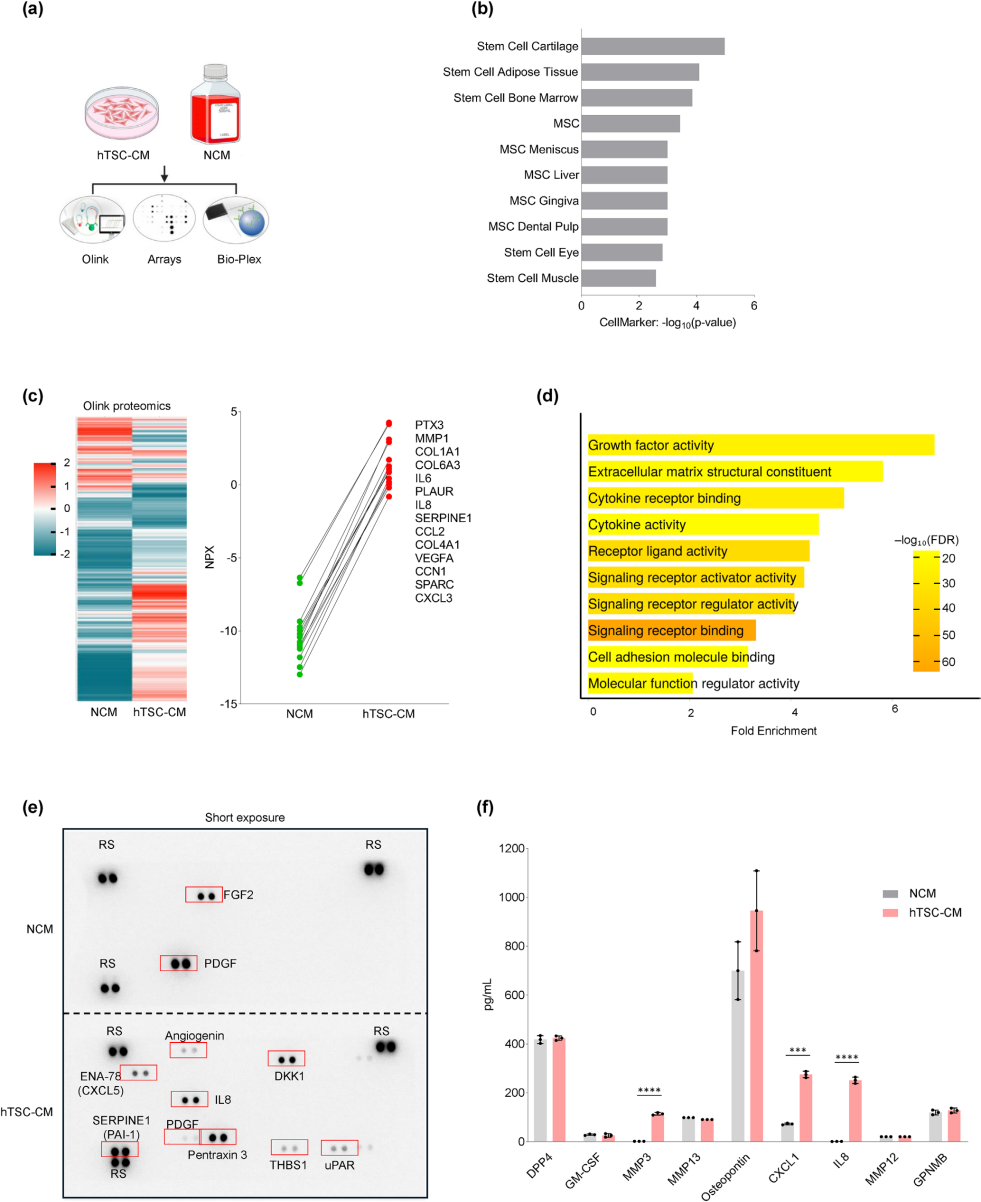
Characterization of the secretome in hTSC‐CM. (a) The proteins present in hTSC‐CM and NCM were quantified by Olink proteomic, protein array, and Bio‐Plex analyses. (b) CellMarker analysis of protein profiles associated with stem cell types identified from Olink proteomic analysis of hTSC‐CM compared to NCM. (c) Heatmap of the Olink proteomic datasets in NCM and hTSC‐CM (*left*); normalized expression of proteins (NPX) more highly abundant in hTSC‐CM compared to NCM in the Olink dataset (*right*); details in Table S3. (d) GO enrichment analysis of hTSC‐CM proteins. (e) Protein array (human cytokine antibody array) analysis of hTSC‐CM compared to NCM; red rectangles, select differentially abundant proteins (low exposure); see Figure S2a for long exposure. (f) Bio‐Plex analysis to quantify secreted proteins enriched in hTSC‐CM compared to NCM. Data in (f) represent the means ±SEM. Statistical significance (***p < 0.001; ****p < 0.0001) was assessed using Student's *t*‐test.

To understand how hTSC‐CM might suppress the SASP, we comprehensively analyzed the protein content in hTSC‐CM using Olink proteomics, cytokine arrays, and Bio‐Plex multiplex assays, as outlined in Figure 3a. Olink proteomic analysis followed by CellMarker analysis revealed that a number of diverse cell types contributed to the proteome present in hTSC‐CM, including stem cell cartilage, stem adipose cells, and bone marrow stem cells (Figure 3b). This analysis further highlighted the presence of proteins linked to stemness and regenerative potential across diverse tissue types in hTSC‐CM. Olink analysis revealed several proteins that were abundant in NCM and reduced in hTSC‐CM (blue), suggesting that they were eliminated or internalized by hTSCs, and several other proteins that were enriched in the hTSC secretome (red), suggesting that they were actively secreted or less internalized by hTSCs (Figure 3c, *left*); the latter group included proteins PTX3, MMP1, COL1A1, COL6A3, IL6, IL8, CXCL3, and VEGFA (Figure 3c, *right*; Table S3). Functional analysis of the hTSC secretome identified pathways such as growth factor activity, extracellular matrix, cytokine receptor binding, cytokine activity, signaling, and cell adhesion (Figure 3d). These pathways suggest that the hTSC‐CM can potentially play roles in cell growth and ECM remodeling.

To complement the Olink proteomic analysis, we performed protein array and Bio‐Plex analyses. The protein array analysis confirmed the enrichment of several key proteins, including PTX3, IL8, SERPINE1, and DKK1, in hTSC‐CM (Figure 3e). A longer exposure of the protein array (Figure S2a) indicated additional proteins in hTSC‐CM, such as IGFBP2, MCP‐1, MIF, BSG, and VEGFA. Interestingly, fibroblast growth factor 2 (FGF2) and platelet‐derived growth factor (PDGF) were less abundant in hTSC‐CM than in NCM (Figure 3e, Figure S2a), suggesting that these proteins were degraded or taken up by the cells. Bio‐Plex analysis (Figure 3f) indicated an enrichment of MMP3, CXCL1, and IL8 in the hTSC‐CM, while senescence‐associated secreted factors such as DPP4 and GPNMB remained unchanged, indicating selectivity in the protein composition in hTSC‐CM. A summary of proteins enriched in hTSC‐CM in the protein array and Bio‐Plex assays is presented in Figure S2b. Together, these results characterize the protein content of the hTSC‐CM secretome, highlighting protein profiles related to stemness and to potential roles in cell growth and tissue repair.

### 2.4 hTSC‐EVs Suppress SASP mRNAs

**FIGURE 4:**
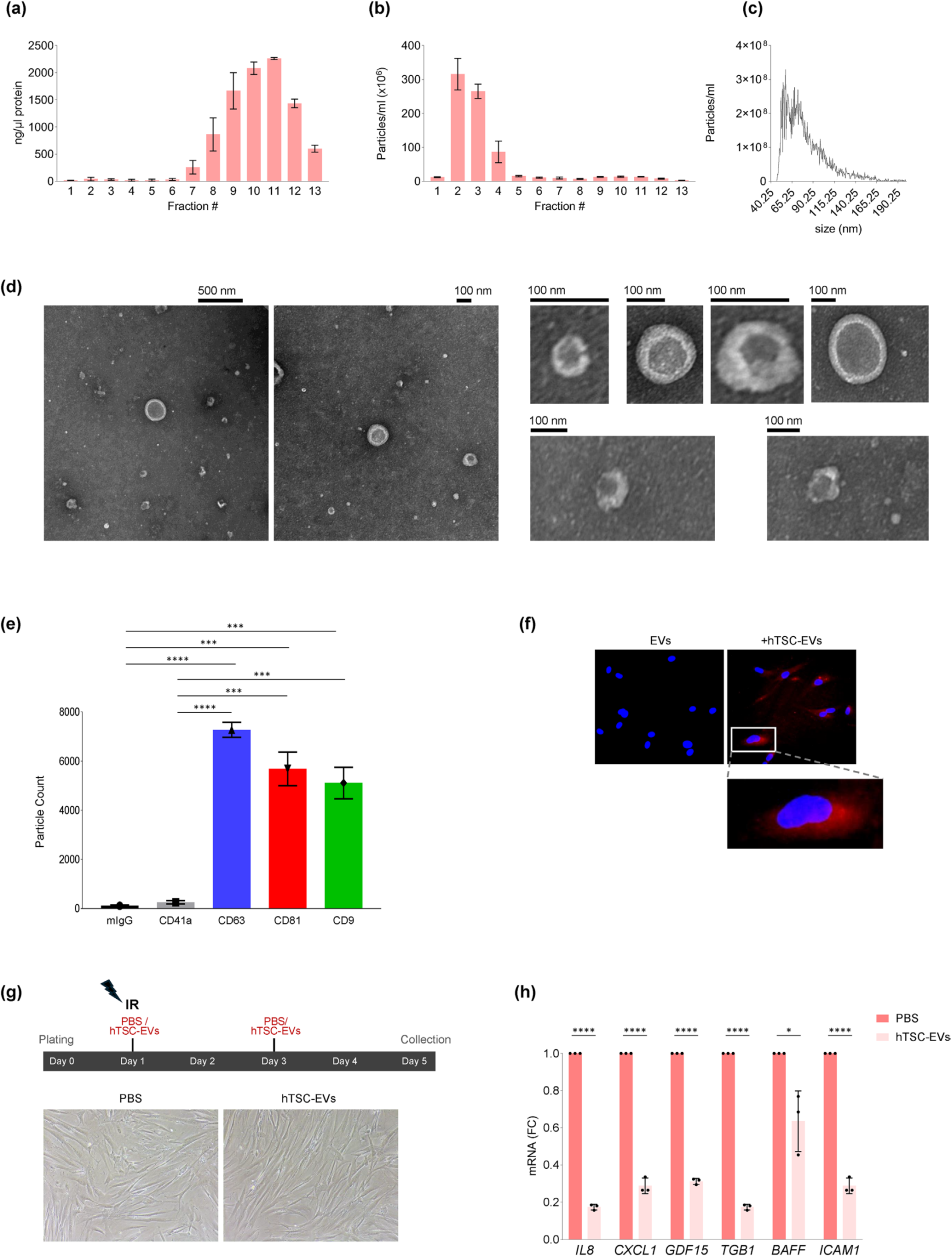
Characterization of EVs in hTSC‐CM and impact on the levels of SASP mRNAs in senescent WI‐38 fibroblasts. (a) Size exclusion chromatography (SEC) profile of hTSC‐CM, showing protein concentration peaks in fractions 7 through 13. (b) SEC elution profile of hTSC‐EVs, identifying EV‐enriched fractions 2 through 4. (c) Nanoparticle tracking analysis (NanoFCM) of hTSC‐EV size distribution and particle concentration, confirming a size range of 40–150 nm. (d) Transmission electron microscopy (TEM) images of hTSC‐EVs, displaying spherical, membrane‐enclosed vesicles. (e) ExoView analysis of hTSC‐EV particle count with surface markers CD63, CD81, and CD9. (f) IR‐treated WI‐38 cells were incubated for 24 h with PKH26‐labeled EVs derived from hTSCs‐CM. Red fluorescence, PKH26‐labeled hTSC‐EVs in WI‐38 fibroblasts. White box, magnified view of a single cell. (g) *Top*, experimental timeline and workflow of WI‐38 cells irradiated (10 Gy) on Day 1 and immediately cultured with purified hTSC‐EVs at 10^10^ particles/mL (or with control vehicle, PBS); fresh hTSC‐EVs or PBS were added on Day 3 and cells were harvested on Day 5 after IR for RNA analysis. *Bottom*, representative micrographs of IR‐treated WI‐38 cells 5 days after incubation with hTSC‐EVs or PBS, to ascertain cell morphology and density. (h) Levels of SASP‐related mRNAs in IR‐treated WI‐38 cells incubated with hTSC‐EVs or PBS; RT‐qPCR analysis was performed on RNA collected on Day 5 after IR, and data were normalized to *GAPDH* mRNA levels. Data in (a, b, e, h) represent the means ±SEM. Statistical significance (*p < 0.05; ***p < 0.001; ****p < 0.0001) was assessed using Student's *t*‐test.

As described above, treatment with the hTSC‐derived secretome (CM), which includes both EVs and soluble non‐EV factors, led fibroblasts undergoing senescence to express reduced levels of SASP‐related mRNAs and to secrete lower concentrations of SASP factors. To dissect the specific contribution of hTSC‐EVs, we isolated EVs from the hTSC secretome using size exclusion chromatography (SEC). Fractions 7 to 13 were enriched in soluble proteins, as shown by the protein concentration peaks in the SEC chromatogram (Figure 4a), while fractions 2 to 4 were largely devoid of soluble proteins and instead contained EVs, as identified by their distinct elution profile (Figure 4b). These EV‐enriched fractions (fractions 2 to 4) were pooled, concentrated using a 3‐kDa cutoff filter, and analyzed for size distribution and particle concentration using NanoFCM, which confirmed particle sizes distributing mainly in the range of 50–150 nm, consistent with the sizes of small EVs such as exosomes (Figure 4c). The identity and morphology of the isolated hTSC‐EVs were confirmed using transmission electron microscopy (TEM), which identified spherical, membrane‐enclosed vesicles with diameters averaging ~80 nm, exhibiting a well‐defined lipid bilayer typical of EVs (Figure 4d). Further characterization of the surface markers of EV‐associated proteins using an ExoView instrument detected a robust presence of canonical EV markers CD63, CD81, and CD9 (Figure 4e). The negligible mIgG signal confirmed the specificity of the assay, while the low CD41a signal indicated minimal contamination from platelet‐derived particles (Figure S3a); individual marker channels revealed the heterogeneity in marker expression (Figure S3b). To assess EV uptake, WI‐38 fibroblasts were incubated for 24 h with PKH26‐labeled hTSC‐EVs; uptake was confirmed by intracellular fluorescence (Figure 4f).

To evaluate the functional impact of hTSC‐EVs on the SASP, WI‐38 fibroblasts were irradiated with 10 Gy and immediately afterwards they were incubated with hTSC‐EVs at a concentration of 10^10^ particles/mL (Day 1). EV treatment was refreshed once 48 h later (Day 3), as outlined in Figure 4g, and cells were harvested on Day 5 after IR to carry out RNA analysis. Phase‐contrast micrographs indicated that culture with hTSC‐EVs did not significantly alter cell morphology or density compared to untreated, irradiated control cells (Figure 4g *bottom*). However, RT‐qPCR analysis revealed a significant reduction in the levels of mRNAs encoding SASP factors, including *IL8*, *GDF15*, and *CXCL1* mRNAs, in hTSC‐EV‐treated cells compared to controls (Figure 4h). These findings support the notion that hTSC‐EVs effectively reduce the levels of SASP‐associated mRNAs in recipient cells, suggesting a potential role in mitigating inflammation and tissue remodeling associated with cellular senescence.

### 2.5 hTSC‐EVs Are Enriched in Proteins Downregulated in Senescence

**FIGURE 5:**
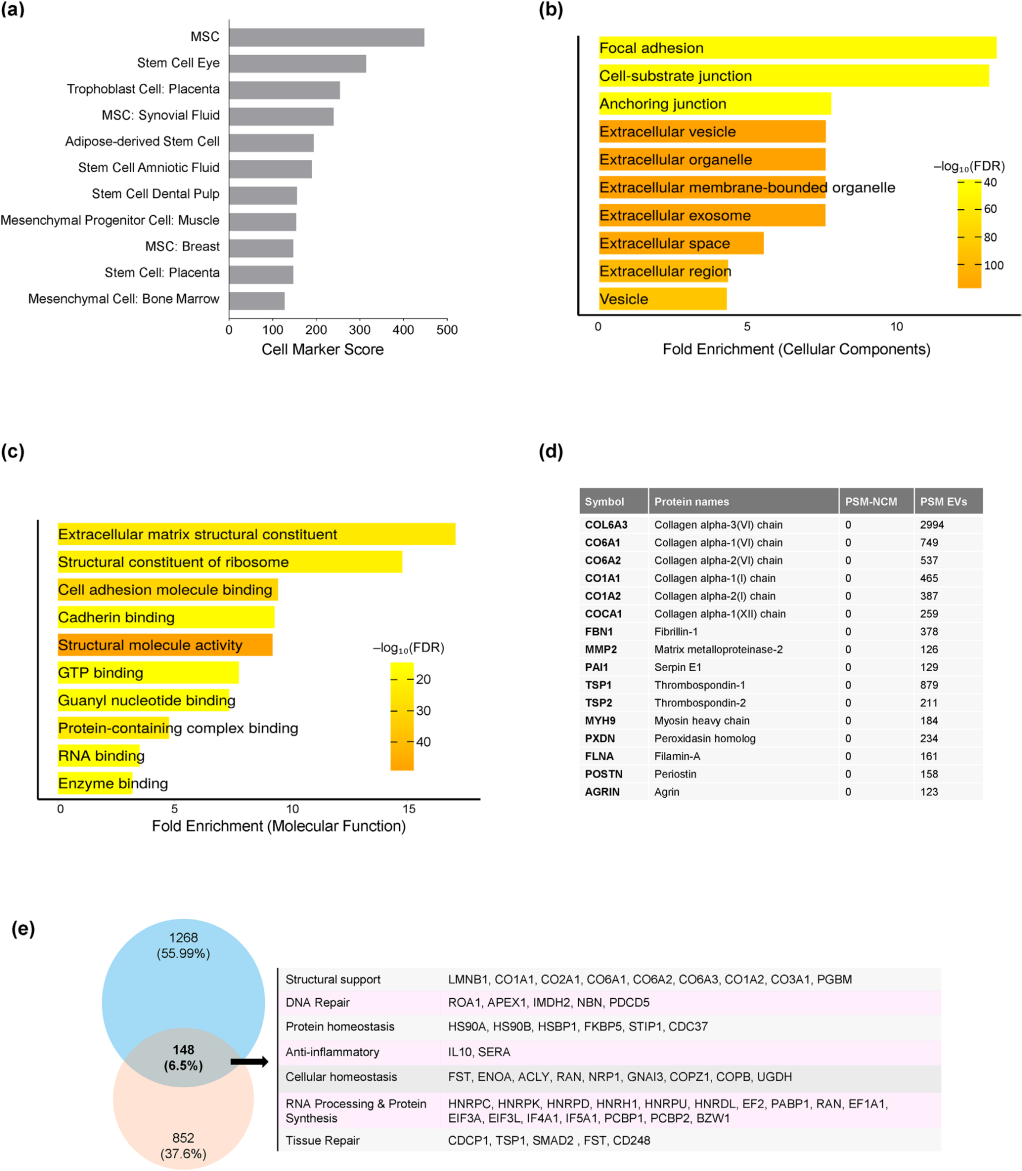
hTSC‐CM and EVs are enriched in proteins downregulated in senescence. (a) CellMarker analysis of the proteome of hTSC‐EVs, showing general enrichment in stem cells and confirming the stem cell origin of hTSCs. (b) GO enrichment analysis of hTSC‐EV cellular components. (c) GO enrichment analysis of hTSC‐EV molecular functions. (d) Mass spectrometry analysis of hTSC‐EV protein composition, highlighting ECM‐related and structural proteins. (e) Venn diagram of protein sets enriched in hTSC‐CM and EVs (pink) and reduced in senescent cells (blue) (Munk et al. 2021); the intersection shows 148 shared proteins (Table S5) including the highlighted proteins, grouped by function.

For a more comprehensive understanding of hTSC‐EVs, we performed mass spectrometry (MS) analysis (MassIVE MSV000096320). The enrichment of stem cell‐derived proteins in the EVs was evaluated by the cell marker score; as shown in Figure 5a, the proteins present in hTSC‐EVs were scored as mesenchymal stem cells, trophoblasts, and placental stem cells, confirming that the hTSC‐EVs analyzed maintained their global stem cell features. The cellular components and structural functions of the proteins identified in the hTSC‐EVs were then examined. GO analysis revealed that these proteins are associated with extracellular organelles, vesicles, and exosomes (Figure 5b), and they are also associated with focal adhesions, cell‐substrate junctions, and anchoring junctions, which are critical for cell adhesion and communication with the surrounding ECM (Figure 5b). Regarding molecular functions, hTSC‐EV proteins were enriched in extracellular matrix structural constituents, suggesting roles in ECM maintenance and stability (Figure 5c). MS analysis further identified a range of collagen types and ECM‐related proteins, such as Collagen alpha‐3(VI) chain, MMP2, Thrombospondin‐1 (TSP1), and Agrin (Figure 5d, Table S4).

Next, we integrated data from the Olink and mass spectrometry (MS) analyses to identify proteins present in both the EV and non‐EV fractions of the hTSC secretome, and we examined their relevance to cellular senescence. Interestingly, we found 148 proteins enriched in the hTSC secretome (Figure 5e, Table S5) that were previously reported to be downregulated in senescent cells (Munk et al. 2021), including Lamin B1 (LMNB1) and Heterogeneous nuclear ribonucleoprotein C (HNRNPC), which play roles in nuclear structure and RNA processing, respectively. These findings suggest that hTSC‐EVs contain proteomes rich in stem cell‐ and ECM‐related factors and may potentially restore proteins that are lost or reduced in senescent cells.

### 2.6 hTSC‐CM and EVs Suppress DNA Damage and NF‐κB Activity

In senescence, DNA damage (as triggered by IR or Eto) is tightly interconnected with signaling leading to the activation of the transcription factor NF‐κB, which in turn amplifies the SASP. We thus hypothesized that hTSC‐CM and EVs may broadly impact senescence by interfering with NF‐κB activation.

**FIGURE 6:**
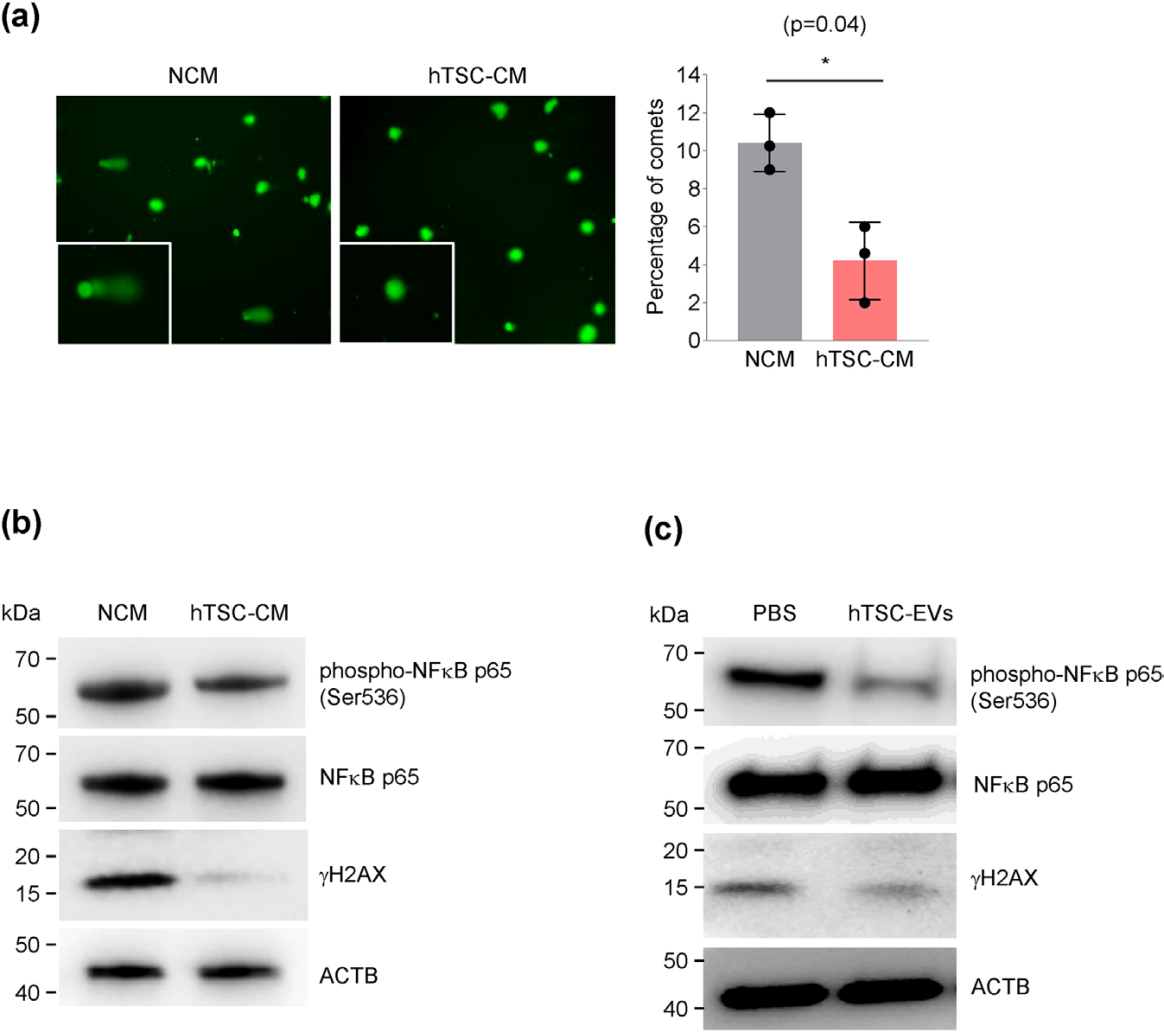
hTSC‐CM and EVs suppress DNA damage and NF‐κB activity. (a) Comet assay images (left) and quantification of DNA damage (graph) in IR‐treated WI‐38 cells that were incubated with NCM or hTSC‐CM following irradiation (10 Gy) for 5 days. (b) Western blot analysis of the levels of phospho‐NF‐κB p65 (Ser536), total NF‐κB, γH2AX, and ACTB (β‐Actin) in IR‐treated WI‐38 cells that were incubated with NCM or hTSC‐CM for 5 days. (c) Western blot analysis of the levels of phospho‐NF‐κB p65 (Ser536), total NF‐κB, γH2AX, and ACTB in IR‐treated WI‐38 cells that were incubated with hTSC‐EVs or PBS for 5 days. Data in (a) represent the means ±SEM. Statistical significance (*p < 0.05) was assessed using Student's *t*‐test.

To assess DNA damage in WI‐38 cells after IR, we performed the Comet assay, which measures the extent of unrepaired DNA in the form of fast‐migrating tails (comets). In IR‐treated cells that were incubated with NCM, comets showed longer and brighter tails, indicating higher levels of DNA damage. In contrast, IR‐treated cells that were incubated with hTSC‐CM showed shorter, less intense tails, suggesting reduced DNA damage (Figure 6a). Following IR treatment of WI‐38 cells, incubation with NCM led to higher levels of phospho (γ)H2AX (a marker of DNA damage) and phospho (p)NF‐κB (a marker of NF‐κB activation) compared to the levels in WI‐38 cells incubated with hTSC‐CM or EVs, which exhibited lower γH2AX and p‐NF‐κB signals, suggesting reduced DNA damage and reduced NF‐κB activation (Figure 6b). Similarly, treatments with hTSC‐EVs revealed decreased γH2AX and p‐NF‐κB in senescent WI‐38 cells (Figure 6c). These findings indicate that hTSC‐CM and hTSC‐EVs suppress or repair DNA damage and suppress NF‐κB activation, culminating in decreased SASP factor production (Figures 2, 4, and 6b,c).

## 3 Discussion

Cellular senescence is a double‐edged sword in aging and disease (Calcinotto et al. 2019). While it protects against tumorigenesis and facilitates tissue repair after acute damage, the accumulation of senescent cells in organs drives age‐related diseases through SASP‐associated inflammation, fibrosis, and tissue dysfunction (Kumari and Jat 2021). To alleviate these detrimental effects, several therapeutic strategies have been developed targeting senescent cells, including senolytics, which induce apoptosis of senescent cells, and senomorphics, which suppress the SASP (von Kobbe 2019). Senolytics, such as dasatinib and quercetin, have demonstrated efficacy in reducing the senescent‐cell burden and alleviating age‐related symptoms in preclinical models (Lelarge et al. 2024). Senomorphics, targeting pathways like NF‐κB, JAK/STAT, and mTOR, include compounds like metformin and rapamycin, which reduce inflammation and mitigate SASP‐related damage (Salminen et al. 2012). Recent studies suggest that combining senolytics and senomorphics may enhance therapeutic efficacy while minimizing side effects, offering promising avenues to improve health span and combat chronic conditions associated with aging (Luis et al. 2022; Robbins et al. 2021).

Stem cells exhibit significant regenerative and therapeutic potential across a range of diseases, primarily through their paracrine activity, which involves the secretion of bioactive factors such as cytokines, growth factors, and microRNAs. For example, MSCs secrete soluble factors like vascular endothelial growth factor (VEGF), transforming growth factor‐beta (TGF‐β), and interleukin‐10 (IL10), which regulate inflammation and promote tissue regeneration (Bagno et al. 2022; Fan et al. 2020; Merimi et al. 2021). In addition, MSC‐derived EVs, including exosomes, can recapitulate the therapeutic effects of their parent MSCs by delivering genetic materials to target cells, thereby regulating gene expression and cell function. MSC‐secreted molecules create a pro‐regenerative microenvironment that supports tissue repair, modulates immune responses, and promotes angiogenesis (Bagno et al. 2022; Han et al. 2022). Unfortunately, the broad use of MSCs is hampered by concerns related to proliferation capacity, differentiation, targeting desired cells, and safety; MSC‐EVs also present challenges in production scaling and functional efficacy (Kou et al. 2022; Lee 2018; Wang et al. 2020).

Given these limitations, hTSCs and their secretome, including EVs, present promising alternatives and offer advantages for regenerative medicine thanks to their ability to remain proliferative and differentiate into various cell types (Chang and Parast 2017; Dong et al. 2020). In addition, EVs derived from hTSCs exhibit regenerative properties on MSCs, making them promising candidates for therapeutic interventions (Go et al. 2023). However, the impact of the hTSC‐CM and EVs on senescence, aging, and age‐related diseases is unknown.

In this study, we present evidence that the secretome and EVs of hTSCs exert potent anti‐senescence effects upon irradiated WI‐38 human lung fibroblasts, particularly by suppressing the production of SASP‐related mRNAs and proteins (e.g., CXCL1, IL8, and GDF15). Analysis of the hTSC‐CM and EV proteomes revealed enrichment of proteins involved in ECM remodeling (e.g., COL1A1 and MMP1) and growth factors (e.g., VEGFA); these proteins are implicated in pathways that promote ECM organization, proliferation, and tissue repair, suggesting that hTSC‐CM may contribute to a regenerative microenvironment (Williams and Ehrhart 2022). The ECM‐modulating and stemness properties observed in MSC‐EVs further support this notion (Kou et al. 2022; Patel et al. 2023). The fact that proteins enriched in hTSC‐CM and EVs (e.g., VIME, HNRNPC, HSP90, ENO1, ACLY, PABP1, HNRNPK, EF2, IMDH2, RAN, PPIA, ROA1, and TSP1) are often downregulated in senescent cells (Munk et al. 2021) suggests that hTSC‐CM and EVs may elicit their beneficial effect at least in part by restoring these depleted factors in senescent fibroblasts. In this regard, through transfer from hTSC‐CM or hTSC‐EVs to senescent cells, VIME could restore cytoskeletal integrity (Patteson et al. 2021), HNRNPC may restore RNA processing (Niggl et al. 2023), and HSP90 may mitigate stress and promote homeostasis (Somogyvári et al. 2022). Further studies are needed to validate their cargo specificity and the underlying mechanisms of action.

In line with growing interest in stem cell‐derived secretomes and EVs as modulators of senescence and aging, we found that culturing irradiated WI‐38 cells with hTSC‐CM or EVs reduced the production of many SASP factors (e.g., IL1A, IL6, and MMP3) and the mRNAs that encode them. Furthermore, the presence of ECM structural components and cell adhesion factors in hTSC‐EVs may contribute to stabilizing the cellular microenvironment and counteracting SASP‐driven matrix degradation (Basisty et al. 2020; Patel et al. 2023). While the exact mediators await identification, proteomic analysis suggests that hTSC‐EVs act through mechanisms involving the suppression of SASP‐associated inflammation and ECM remodeling.

Functional analysis indicated that both hTSC‐CM and EVs reduced DNA damage and NF‐κB phosphorylation, two inducers of the SASP (Figure 6), suggesting that hTSC‐CM and EVs are promising senomorphic targets. The observed reduction in γH2AX, a robust marker of DNA damage, suggests that hTSC‐CM and EVs may enhance DNA repair capabilities or deliver protective factors that mitigate genotoxic stress. In this regard, EVs were previously reported to modulate biological processes relevant to age‐related changes in hematopoietic stem cell (HSC) phenotypes, including DNA damage responses (Goldberg 2021); our results (Figure 6c) suggest that hTSC‐EVs alone might be sufficient to elicit anti‐senescence traits. Furthermore, a recent study found that MSC‐derived apoptotic vesicles (apoVs) can modulate DNA damage responses and repair mechanisms in recipient cells, rescuing DNA damage and premature senescence in mice with apoptosis deficiencies. These apoVs were found to contain multiple nuclear DNA repair enzymes, including PARP1, which were transferred to recipient cells to alleviate DNA damage, indicating a potential role for EVs as carriers of functional repair proteins, and offering a novel therapeutic path for diseases of genomic instability (Huang et al. 2024). Interestingly, our proteomic analysis identified several proteins directly involved in DNA repair within the hTSC‐CM and EVs, including CHEK2, NBN, RAD23B, PARP1, PMS1, and APEX1 (Scheijen and Wilson 2022).

The reduction in phospho‐NF‐κB p65 (Ser536) in cells treated with hTSC‐CM or EVs suggests a suppression of inflammatory signaling, likely through DNA damage, which is known to trigger NF‐κB transcriptional activity to increase transcription of pro‐inflammatory genes (Giridharan and Srinivasan 2018; Budamagunta et al. 2021; Chien et al. 2011; Vaughan and Jat 2011). Persistent activation of NF‐κB leads to chronic inflammation, which promotes biological aging and age‐related diseases (Garcia‐Garcia et al. 2021). Thus, the ability of hTSC‐CM and EVs to attenuate NF‐κB activity indicates that these treatments may mitigate the inflammatory responses exacerbated by DNA damage, and aligns with other evidence that EVs can modulate inflammatory pathways in aging, such as those affecting old HSC phenotypes (Goldberg 2021).

Although we found that incubation with hTSC‐CM/EVs reduced DNA damage and NF‐κB activation in senescent fibroblasts, the molecular mechanisms underlying these effects are unclear. Further research is needed to identify the mechanisms through which hTSC‐CM/EVs elicit their anti‐senescence effects. The proteomic data in Figure 5c suggest that we focus on mechanisms centered around (i) DNA damage sensing and repair, (ii) proteostasis, (iii) nuclear lamina and RNA processing, and (iv) ECM and focal adhesion signaling. Additionally, it is unclear whether specific proteins within hTSC‐CM and EVs (e.g., HNRNPC and HSP90) actively restore function in cells or contribute to a more favorable microenvironment. Future investigation of the RNAs (mRNAs, microRNAs, and long noncoding RNAs) in hTSC‐EVs and their roles in recipient cells is also likely to offer valuable insight.

An additional limitation of our study stems from the identity and state of the hTSC lines used as the source of the secretome and EVs. Although these lines were derived from pre‐placental chorionic villi and maintained stable morphology and expected marker expression over serial passages, we could not fully exclude the possibility that the culture conditions favored expansion of chorionic mesenchymal stromal cells and/or promoted partial trophoblast differentiation. Because the MesenCult‐ACF conditions used here were optimized for MSC‐like lineages and not for maintaining canonical hTSC stemness, future work should incorporate trophoblast‐specific media (Okae et al. 2018), together with hTSC‐defining transcriptomic benchmarks and lineage‐specific sorting strategies to more rigorously verify and maintain the trophoblast stem‐cell state. These steps will help distinguish trophoblast‐specific effects of the secretome and EVs from those potentially mediated by other early‐gestation progenitor populations.

Likewise, identifying the receptors and surface proteins on recipient cells that mediate the effects of hTSC‐CM and EVs is essential for understanding the specificity of their actions. Our study focused on WI‐38 fibroblasts but not other cell types. Given the heterogeneity of senescence across different tissues and pathological contexts, future studies should also assess the effects of hTSC‐CM and EVs in other senescent models, including endothelial, immune, and epithelial cells. Furthermore, while our findings in culture suggest a therapeutic potential for hTSC‐CM and EVs in mitigating senescence, scaling up the production of hTSC‐CM and EVs will allow us to explore their efficacy in animal models. Future studies should investigate the effects of hTSC‐CM and EVs on old mice or disease‐specific mouse models (e.g., osteoarthritis, pulmonary fibrosis, or Alzheimer's disease) to assess their impact on systemic senescence, SASP factors in circulation, health span, and longevity. Moreover, comparative analyses between hTSC‐CM/EVs and other stem cell‐derived secretomes/EVs will be crucial to determine whether hTSC‐CM/EVs offer distinct or superior benefits in antiaging and regenerative applications. Finally, while the placental origin of trophoblast stem cells may uniquely reduce immune rejection and enhance therapeutic efficacy, it is not known if this advantage is also seen with the secretome or EVs. Thus, the immunogenicity, biodistribution, and long‐term safety of hTSC‐CM and EVs in preclinical and clinical settings remain to be rigorously evaluated.

In summary, we have found that hTSC‐CM and EVs elicit anti‐senescence effects by attenuating DNA damage and NF‐κB signaling, in turn suppressing the inflammatory cascades that amplify SASP production and secretion. These findings underscore the potential value of hTSC‐derived secretomes and EVs as senotherapeutics to improve health span.

## 4 Methods

### 4.1 Cell Culture, Treatments, and SA‐β‐Gal Activity

Human WI‐38 diploid fibroblasts (Coriell Institute for Medical Research: ID AG06814) were grown in Dulbecco's Modified Eagle Medium (DMEM) supplemented with 10% fetal bovine serum (FBS), 1% non‐essential amino acids, and 1% antibiotics (Gibco). Cells were maintained at 37°C in a humidified atmosphere with 5% CO_2_ and routinely tested negative for mycoplasma. Proliferating WI‐38 fibroblasts (between PDLs 22 and 24) were treated with a single IR dose (10 Gy) on Day 1, and the medium was immediately supplemented with either NCM or hTSC‐CM. On Day 3, the medium was replaced and fresh NCM or hTSC‐CM was added until harvest on Day 5. Etoposide (Eto) treatment (50 μM) started on Day 1 together with NCM or hTSC‐CM, and continued until Day 3, when the medium was replaced with fresh Eto and supplemented with fresh NCM or hTSC‐CM. All samples were collected on Day 5 to assess the impact of hTSC‐CM (relative to NCM) on cells exposed to a senescence trigger (Figure 1c). Senescence‐associated β‐galactosidase (SA‐β‐Gal) activity, a widely used biomarker of senescent cells in culture and in tissues, detects lysosomal β‐galactosidase activity at pH 6.0; this activity is specifically elevated in senescent cells due to the increase in lysosomal mass and function that accompanies the senescent phenotype. The pH‐dependent detection distinguishes senescent cells from proliferating cells, as normal lysosomal β‐galactosidase activity is optimal at pH 4.0. SA‐β‐Gal activity was detected as described (Abdelmohsen et al. 2024), following the manufacturer protocol (Cell Signaling Technology, cat. #9860). Images of SA‐β‐Gal staining were acquired using a Nikon Digital Sight camera system attached to a Nikon Eclipse TS100 microscope.

### 4.2 hTSC Culture, Flow Cytometry, hTSC‐CM Concentration, and Treatment of Fibroblasts

Human trophoblast stem cells ABX‐001 hTSC 2003 were originally derived from chorionic villi obtained during salpingectomy/salpingotomy procedures for ectopic pregnancy early in gestation. Tissue procurement, derivation, and banking were performed under IRB‐approved protocols (KMUHIRB‐F(I)‐20200184) and ethics review by the Stanford Center for Biomedical Ethics (available upon request). Cells were cultured in conditioned media (MesenCult, Stemcell Technologies, Cat#05446) at Accelerated Bioscience with confirmed mycoplasma‐free and pathogen‐free status, demonstrating post‐thaw viabilities of approximately 97%–98%. The morphology of hTSC cells was documented by imaging in adherent culture using a Keyence (BZ‐X710) phase‐contrast microscope equipped with a 4× objective.

The presence of surface markers in hTSCs was evaluated by flow cytometry using a CytoFLEX flow cytometer (Beckman Coulter). Cells were harvested, counted, and stained for 30 min with either PE‐conjugated primary antibodies recognizing CD45, CD90, and CD105 (R&D Systems) or corresponding isotype controls (R&D Systems; 500,000 cells/sample). Data analysis and measurement of percentages of positive and negative cells was performed using the FlowJo software (BD).

Conditioned medium from passages 1 to 11 was collected from hTSC cultures to test its impact on cellular senescence. Additionally, EVs were isolated from the conditioned media to evaluate their effects on senescence phenotypes. For the control non‐conditioned media (NCM), MesenCult was used directly as supplied by the manufacturer, without culture of any cells. All media handling was performed in a sterile cell culture environment, followed by centrifugation to remove cellular components and debris. Cell culture media were concentrated from 200 mL to 10 mL using a 3‐kDa molecular weight cutoff filter (Amicon Ultra‐15, UFC900308) by centrifugation at 3000 RPM for 1 h at 4°C; after it was concentrated 20×, the medium was stored at −80°C until use. WI‐38 cells were incubated with a final 2× secretome immediately following IR treatment or concurrently with Eto treatment; the media were refreshed including Eto at regular intervals as shown in Figure 1c. Similarly, WI‐38 cells were incubated with the hTSC‐EVs immediately following IR treatment and the media were refreshed with EVs on Day 3 as shown in Figure 4g.

### 4.3 Size Exclusion Chromatography (SEC) and Culture With hTSC‐EVs

EVs were isolated from the concentrated hTSC‐CM (secretome, as explained above) using qEV10, 35‐nm SEC columns (IZON Science), according to the manufacturer protocol. Briefly, 10 mL of secretome concentrate was loaded onto the column, after which 13 fractions (5 mL each) were collected to assess EV and protein distribution. Typically, the first 2 to 4 fractions contained EVs, which were then concentrated using a 3‐kDa molecular weight cutoff filter. The size and distribution of the concentrated EV sample were subsequently assessed using a NanoFCM instrument. Cells were treated with EVs (~10^9^ particles/mL) in 24‐well plates.

### 4.4 EV Labeling and Uptake

EVs were labeled with the red fluorescent lipid dye PKH26 (Sigma‐Aldrich, St. Louis, MO) with modifications to minimize dye micelle formation. Briefly, 100 μL of EVs (10^10^ EVs/mL) were mixed with 0.2 μM PKH26 solution in diluent C (Sigma‐Aldrich) and incubated for 1 min at room temperature. As a control, 100 μL of PBS alone were subjected to the same PKH26 staining procedure to assess potential micelle formation. After labeling, 100 μL of PBS were added to the EV solution, and the free dye was removed using a Zeba Spin Desalting Column (7K MWCO, Thermo Fisher Scientific, cat. # 89892). To assess EV uptake, WI‐38 cells were incubated with PKH26‐labeled hTSC‐EVs for 24 h at 37°C and 5% CO_2_. Following incubation, cells were washed twice with PBS to remove unbound EVs and fixed with 3% formaldehyde in PBS for 10 min at RT. After fixation, cells were permeabilized with 0.1% Triton X‐100 (Sigma‐Aldrich) in PBS for 5 min at room temperature, then washed 3× with DPBS to remove residual Triton X‐100. To visualize nuclei, cells were stained with DAPI and fluorescent images were captured using a Keyence microscope.

### 4.5 Nanoflow Cytometry (NFCM)

The size distribution of the isolated EVs was analyzed using nanoflow cytometry (NFCM) as previously described (Abdelmohsen et al. 2023) with the Flow NanoAnalyzer (NanoFCM Inc.). Briefly, the instrument was calibrated using 250‐nm Silica Beads to establish particle concentration and a Silica Nanosphere Cocktail to determine particle size. Particle concentration and size were measured based on the calibration curve, flow rate, and side scatter intensity, with data acquisition performed for 1 min per sample, following the manufacturer guidelines for NanoFCM operation.

### 4.6 ExoView Analysis

Surface marker proteins on EVs were analyzed using the ExoView R200 platform (NanoView Biosciences) as described (Abdelmohsen et al. 2023). Briefly, 50 μL of EV samples at equal concentrations were incubated on ExoView Tetraspanin chips in a 24‐well plate for 16 h. Following incubation, the chips were washed three times with 1× Solution A before adding 250 μL of detection antibodies in blocking buffer for 1 h. Subsequent washing steps included two washes with Solution A, three washes with Solution B, and a final rinse with deionized water. After drying on absorbent paper, the chips were scanned using the ExoView R200 system and analyzed with ExoScan software (NanoView Biosciences).

### 4.7 Transmission Electron Microscopy

For transmission electron microscopy (TEM) imaging analysis (Johns Hopkins University EM facility), 20 μL of EV samples (approximately 10^10^ particles/mL) were placed onto carbon‐coated parlodion copper grids and allowed to adhere for 2 min. The grids were then floated sequentially on two drops of filtered 0.75% uranyl acetate aqueous solution containing 0.03% tylose for 1 min each, followed by blotting with filter paper for adsorption. EVs were immobilized on poly‐L‐lysine‐coated coverslips using a fixation solution of 1% glutaraldehyde in 80 mM phosphate buffer with 5 mM MgCl₂. Coverslips were subsequently rinsed with sucrose‐containing buffer and post‐fixed in osmium tetroxide reduced with potassium ferrocyanide for 1 h on ice.

To improve visualization, *en bloc* staining was applied using a 2% uranyl acetate solution in maleate buffer for 1 h. The EV samples were then dehydrated through a sequence of ethanol solutions (ranging from 30% to 100%), then embedded in Eponate 12 resin, and hardened by heating at 60°C for 48 h. Coverslips were subsequently mounted onto inverted beam capsules and immersed in liquid nitrogen for 10 min, then detached from the capsules. The solidified resin blocks were trimmed and ultrathin sections (50–60 nm) were obtained using a Reichert Ultracut E ultramicrotome equipped with a Diatome diamond knife. Sections were stained with methanolic uranyl acetate, followed by lead citrate incubation. Between staining steps, thorough rinsing was performed with distilled water. Coverslips were fixed in 2% glutaraldehyde in 100 mM sodium cacodylate buffer and stained with 2% uranyl acetate for 20 min and periodically rinsed with distilled water. Finally, imaging was performed on a Hitachi H‐7600 TEM operated at 80 kV. Digital images were acquired using an XR50 5‐megapixel CCD camera from Advanced Microscopy Techniques Corp.

### 4.8 Mass Spectrometry Analysis

For mass spectrometry (MS) analysis, proteins were extracted from the isolated EVs, and protein concentrations were determined using the Pierce MicroBCA kit. Samples were processed by treating with DTT for reduction, subsequently with iodoacetamide for alkylation, and finally digested with trypsin in a 25 mM NH_4_HCO_3_ solution. The resulting tryptic peptides were purified using a C18 column before being reconstituted in 25 μL of 0.1% formic acid; 12 μL of this mixture was analyzed by liquid chromatography‐tandem mass spectrometry (LC‐MS/MS) at 110‐min run time (Thermo Scientific Orbitrap Exploris 240 Mass Spectrometer and a Thermo Dionex UltiMate 3000 RSLCnano System). The Orbitrap Exploris 240 operated in a data‐dependent mode, alternating between full‐scan MS and MS/MS acquisition. Peptide mixtures were loaded onto a peptide trap cartridge at a flow rate of 5 μL/min before being transferred onto a Thermo Fisher Scientific EasySpray C18 column via a linear gradient of 3%–36% acetonitrile in 0.1% formic acid over 110 min at a flow rate of 0.3 μL/min. Ionization was performed using a Nano‐EasySpray Ion Source with a spray voltage of 1.6 kV and a capillary temperature of 275°C. Eluted peptides were ionized and sprayed into the mass spectrometer using a Nano‐EasySpray Ion Source (Thermo Fisher Scientific) with spray voltage of 1.6 kV and capillary temperature of 275°C. The instrument was set to isolate and fragment the 15 most intense multiply charged ions (z ≥ 2) using higher‐energy collisional dissociation (HCD) with a normalized collision energy of 30. The AGC target was adjusted to 10^5^, with a maximum injection time of 200 ms and a resolution of 17,500. The isolation window was set to 2, and a dynamic exclusion of 20 s was applied to minimize redundant fragmentation. Charge state screening was enabled to exclude unassigned ions and those with a charge state of 1+ or ≥ 7+. To ensure specificity, the same steps were performed using MesenCult (Stemcell Technologies, Cat#05445) as a negative control. Raw data files were processed using Proteome Discoverer (v1.4) and analyzed via the SEQUEST algorithm (Thermo Fisher Scientific). The acquired spectra were searched against protein sequence database from UniProtKB/Swiss‐Prot. Peptide filtering criteria included a minimum sequence length of five amino acids and a maximum false discovery rate (FDR) threshold of 0.01. Assembled proteins with peptide spectrum match (PSM) counts were quantified and normalized using the normalized spectral abundance factors (NSAFs) to determine their relative abundance. Raw files are available in MSV000096320 [doi: 10.25345/C5QR4P30R].

### 4.9 Protein Extraction and Western Blot Analysis

To extract total protein, cells were rinsed twice in 1× PBS before lysis in a 2% SDS buffer containing 50 mM HEPES and freshly prepared protease/phosphatase inhibitors (Cell Signaling Technology). Protein concentration was quantified with the BCA assay kit (Pierce). The lysates were mixed with 4× SDS Laemmli buffer (Bio‐Rad) supplemented with β‐mercaptoethanol and heated at 95°C for 5 min to denature proteins. For western blot analysis, protein samples were loaded onto Tris‐Glycine gels (Bio‐Rad), separated by SDS‐PAGE, and transferred to nitrocellulose membranes using the iBlot kit (Invitrogen). Membranes were blocked for 1 h at room temperature with 5% nonfat milk in 1× TBST, followed by overnight incubation at 4°C with primary antibodies. Primary antibodies were used that recognized phospho‐Histone H2AX Ser139 (γH2AX) (#9718), phospho‐NF‐κB p65 Ser536 (#3033), and NF‐κB p65 (#8242) (all from Cell Signaling Technology), as well as β‐actin (ACTB) (sc‐8432, Santa Cruz Biotechnology). The membranes were then washed in 1× TBST and incubated with secondary antibodies in 5% nonfat milk for 1 h at room temperature. After washing, membranes were treated with ECL solution (Kindle Biosciences), and chemiluminescent signals were detected using a ChemiDoc imaging system (Bio‐Rad).

### 4.10 RNA Isolation, and RT‐qPCR and RNA‐Sequencing Analyses

Total RNA was extracted from cells using TRIzol reagent (Thermo Fisher Scientific) following the manufacturer's protocol. RNA concentration was assessed using a NanoDrop spectrophotometer (Thermo Fisher Scientific). Reverse transcription (RT) was carried out using 500 ng of total RNA with the Maxima Reverse Transcriptase protocol (Thermo Fisher Scientific) and transcript levels analyzed via quantitative real‐time PCR (qPCR) using the SYBR Green FAST qPCR master mix kit (Kapa Biosystems) on a QuantStudio 5 Real‐Time PCR System (Thermo Fisher Scientific). For qPCR amplification, these specific primer pairs were employed: CAACCAGAGCTGGGAAGATTCG and CCCGAGAGATACGCAGGTGCA for *GDF15*, GAAAGAGTGGCAACCTGCCTTC and GCACCAAGTTTTACTACATCTGCC for *MKI67*, GTCTCCGGGAATCTCTGATGC and GTTCAGTGGAGCCCAGCTCT for *BAFF*, AGTGAGGAACAAGCCAGAGC and GTCAGGGGTGGTTATTGCAT for *IL6*, AGCTTGCCTCAATCCTGCATCC and TCCTTCAGGAACAGCCACCAGT for *CXCL1*, TCCTGATTTCTGCAGCTCTGT and AAATTTGGGGTGGAAAGGTT for IL8, and CATGTACGTTGCTATCCAGGC and CTCCTTAATGTCACGCACGAT for *ACTB* mRNA. RNA levels were calculated with the 2^−ΔΔCt^ method and were normalized to *GAPDH* mRNA levels.

RNA‐Sequencing (RNA‐seq) was conducted on total RNA extracted from WI‐38 recipient cells treated as described in Figure 1a. RNA integrity was evaluated using an Agilent Bioanalyzer, and libraries were generated and sequenced by Azenta Life Sciences. Raw sequencing reads were processed, aligned to the human genome (GRCh38), and subjected to differential expression analysis using DESeq2. Raw sequencing data are available under accession GSE282054.

### 4.11 Cytokine Arrays and Bio‐Plex Analysis

The levels of secreted cytokines in the hTSC conditioned media (CM) or non‐conditioned media (NCM) were assessed using the Human Cytokine Antibody Array (R&D Systems) following the manufacturer protocol, and chemiluminescent signals were captured using a ChemiDoc imaging system (Bio‐Rad).

For multiplex cytokine analysis, the hTSC‐CM or NCM were analyzed using human Luminex Assay kits (R&D Systems) to quantify DPP4, IL8, IL6, GDF15, MMP3, Osteopontin, GM‐CSF, and ICAM1, with media diluted 1:10 in Calibrator Diluent RD6‐52. Standards, blanks, and diluted media were incubated with the microparticle cocktail for 2 h at 25°C, followed by incubation with the Biotin‐Antibody cocktail for 1 h and a final 30‐min incubation with Streptavidin‐PE under shaking conditions at 25°C. Each incubation step was followed by three washes with Wash Buffer, and the plate was analyzed using the Bio‐Rad Bioplex‐200 Instrument with the following settings: 50 μL sample volume, Bio‐Plex MagPlex Beads (Magnetic), Double Discriminator Gates set at 8000–23,000, low RP1 target value for the CAL2 setting, and a bead count of 50 per region. Results were processed and analyzed using Bio‐Plex Manager software (Bio‐Rad).

### 4.12 Olink Proteomics

For Olink proteomic analysis, we used the media to evaluate the secretome of the hTSCs or WI‐38 cells at Indiana University. Briefly, target proteins were detected through a highly specific binding interaction with dual oligonucleotide‐labeled antibody probes. The bound probes underwent microfluidic real‐time PCR amplification, enabling the quantitative detection of the corresponding DNA sequences. Internal and external controls were utilized for quality control and normalization. Normalized Protein expression (NPX) values were provided as the final assay readout corresponding to protein levels.

## Author Contributions

K.A. conceived and supervised the study. K.A., J.L.M., M.R., A.P., M.B., E.M.A., C.H.S., R.M., M.M., C.‐Y.C., and C.J.N.‐O. performed the experiments and collected data. M.S.‐M. and N.D. performed data analysis and statistical evaluations, Y.L., J.‐N.L., S.H.P., and J.H. supplied the hTSC secretome and provided experimental support, K.A. and M.G. supervised and wrote the manuscript with input from all authors. All authors contributed critical feedback that shaped the study and manuscript.

## Funding

This work was supported by National Institute on Aging Intramural Research Program NIH (AG000511).

## Conflicts of Interest

The authors declare no conflicts of interest.

## Supporting information


**Figures S1–S3:** acel70368‐sup‐0001‐FiguresS1‐S3.pdf.

**Table S1:** acel70368‐sup‐0002‐TableS1.zip.

**Table S2:** acel70368‐sup‐0003‐TableS2.zip.

**Table S3:** acel70368‐sup‐0004‐TableS3.zip.

**Table S4:** acel70368‐sup‐0005‐TableS4.zip.

**Table S5:** acel70368‐sup‐0006‐TableS5.zip.

## Data Availability

All data used to generate the statistical analyses and figures in this study are available from the corresponding author upon reasonable request. Raw sequencing data are available at GSE282054. Raw MS data are available at MSV000096320 [doi: 10.25345/C5QR4P30R].
