# Supplementary material for: The Secretome of Human Trophoblast Stem Cells Attenuates Senescence‐Associated Traits
Source: Aging Cell. 2026 Jan 11;25(2):e70368. doi: 10.1111/acel.70368 (PMC12791570; doi:10.1111/acel.70368)
Supplement: Supplementary file 1 — Figures S1–S3: acel70368‐sup‐0001‐FiguresS1‐S3.pdf. [file ACEL-25-e70368-s004.pdf]

Legends of Supplementary Figures

Supplementary Figure 1. (a) Flow cytometry analysis of surface markers of hTSCs at passage 11 (*left*) and 19 (*right*) using antibodies recognizing stem cell markers CD90 and CD105, and the pan-hematopoietic marker CD45 (to identify possible contamination) CD45. **(b)** WI-38 cells were exposed to IR and then incubated with either NCM or hTSC-CM for 5 days, whereupon cell morphology was assessed by phase-contrast microscopy (*left*) and cell number by direct cell counting (*right*). **(c)** RT-qPCR analysis of the levels of *ACTB* and *MKI67* mRNAs, normalized to *GAPDH* mRNA levels. **(d)** WI-38 fibroblasts were treated as in (b) followed by RNA-seq analysis, and GO enrichment analysis was performed on transcripts that were significantly increased in cells incubated with hTSC-CM compared to cells incubated with NCM.

Supplementary Figure 2. (a) Extended exposure of the protein array analysis from human hTSC-CM and NCM, corresponding to Figure 3f. **(b)** Proteins identified using the protein array and Bio-Plex assays comparing hTSC-CM to NCM, corresponding to Figure 3f, 3g, and S2a.

Supplementary Figure 3. **(a)** ExoView images showing the detection of classical EV surface markers CD9, CD63, and CD81, alongside CD41a and IgG controls, in hTSC-EVs. **(b)** Pie charts depicting the proportions of single-, double-, and triple-positive hTSC-EV subpopulations for CD9, CD63, and CD81 markers.

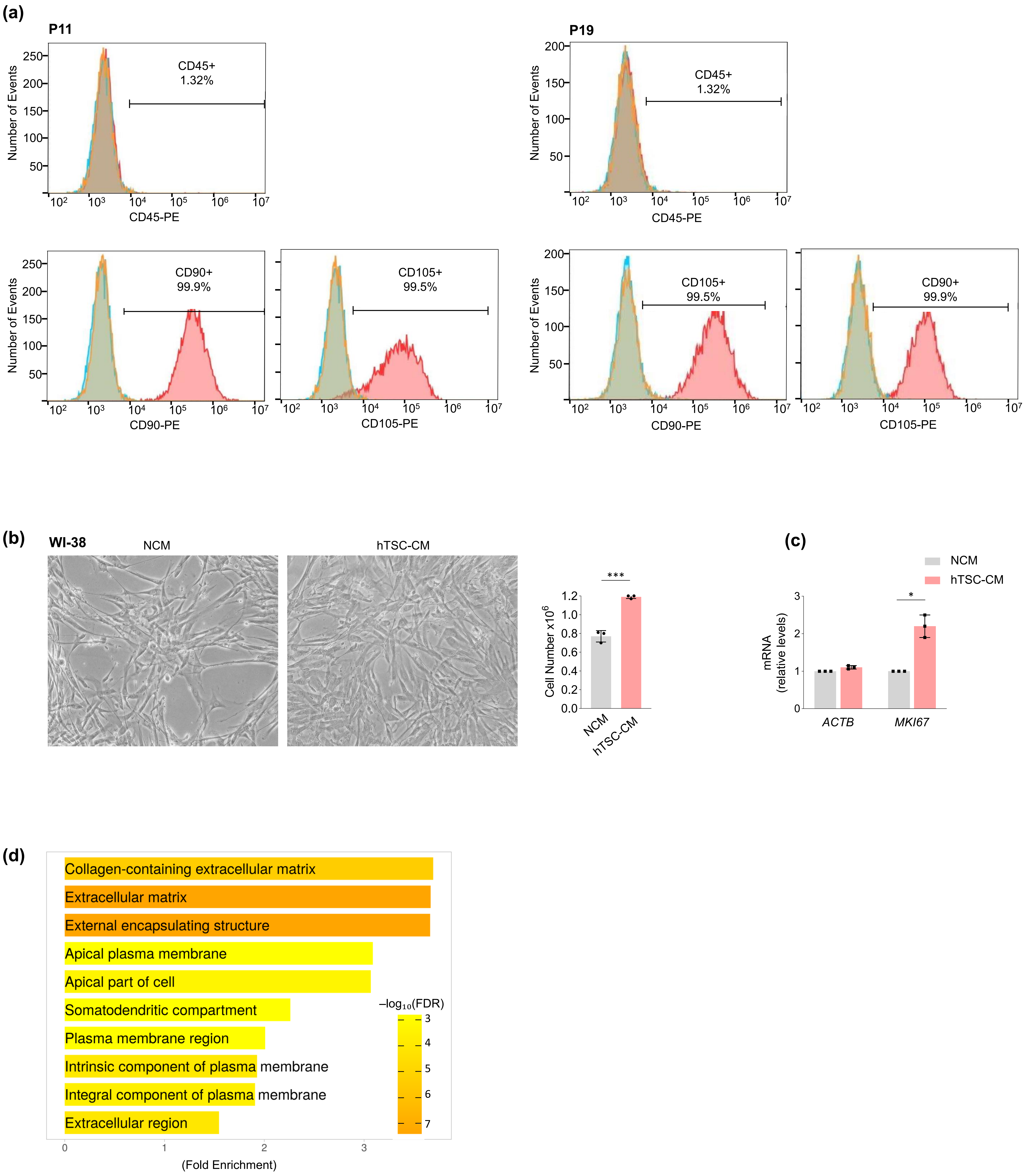
(a)
P11
Number of Events
CD45+
1.32%
CD45-PE
CD90+
99.9%
CD90-PE
CD105+
99.5%
CD105-PE
P19
Number of Events
CD45+
1.32%
CD45-PE
CD105+
99.5%
CD90-PE
CD90+
99.9%
CD105-PE
(b)
WI-38
NCM
hTSC-CM
***
Cell Number x10^6
NCM
hTSC-CM
(c)
NCM
hTSC-CM
*
mRNA (relative levels)
ACTB
MKI67
(d)
Collagen-containing extracellular matrix
Extracellular matrix
External encapsulating structure
Apical plasma membrane
Apical part of cell
Somatodendritic compartment
Plasma membrane region
Intrinsic component of plasma membrane
Integral component of plasma membrane
Extracellular region
−log10(FDR)
(Fold Enrichment)

(a)

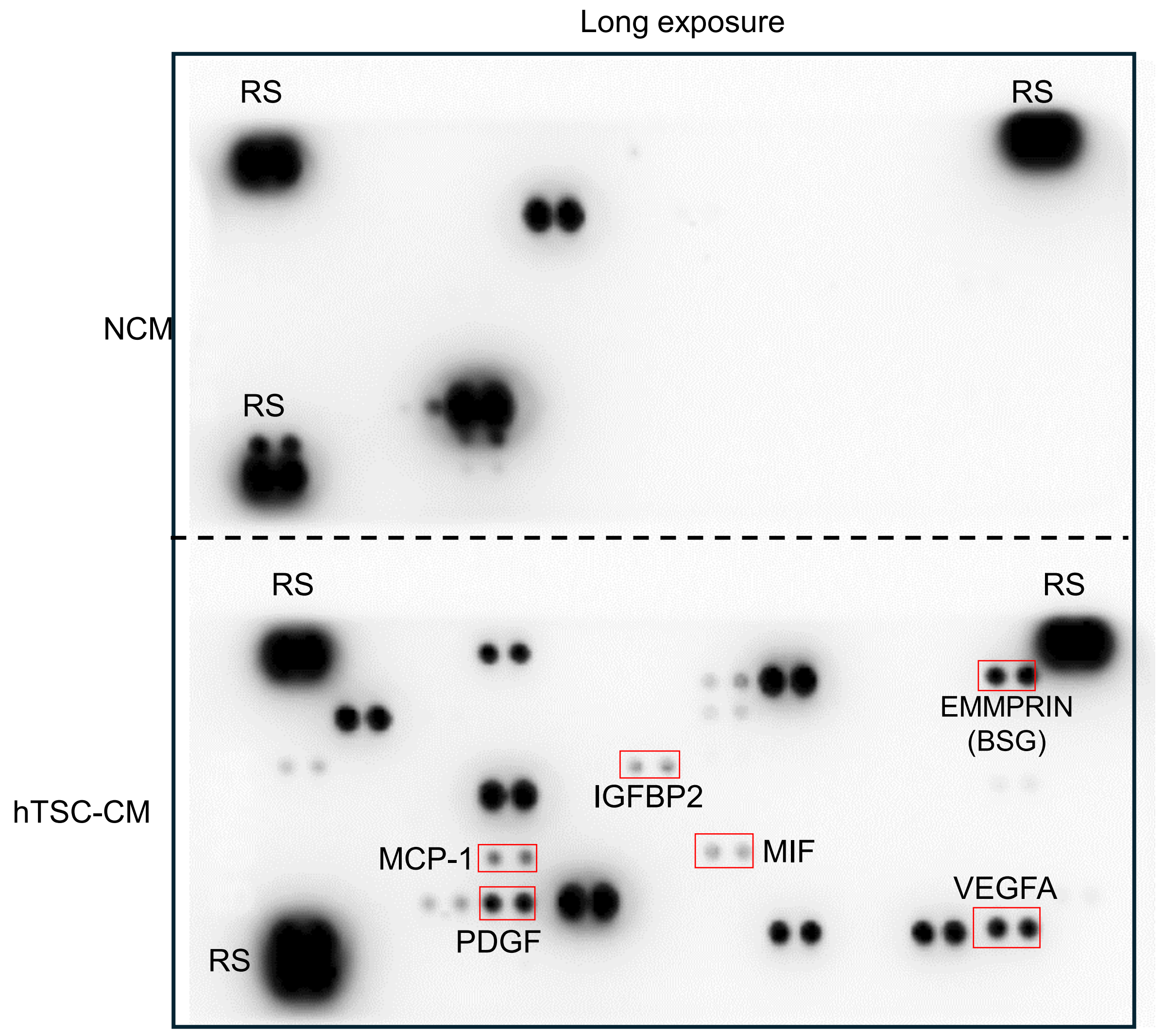

(b)

Protein Name	Functions
ENA-78	Promotes neutrophil recruitment, tissue repair
VEGFA	Enhances vascular repair
SerpinE1	Aids in wound repair, protects ECM during inflammation
Pentraxin 3	Enhances immune defense, tissue repair
THBS1	Facilitates ECM remodeling, wound healing
uPAR	Facilitates cell migration, tissue repair
Dkk-1	Inhibits Wnt signaling, enhances tissue homeostasis
IL8	Enhances neutrophil chemotaxis, tissue regeneration
MCP-1	Promotes wound healing, immune defense.
EMMPRIN	Enhances ECM remodeling, tissue integration
Angiogenin	Stimulates proliferation, migration
CXCL1	Promotes tissue repair
IGFBP-2	Enhances cell survival
MIF	Enhances immune responses, tissue repair

(a)

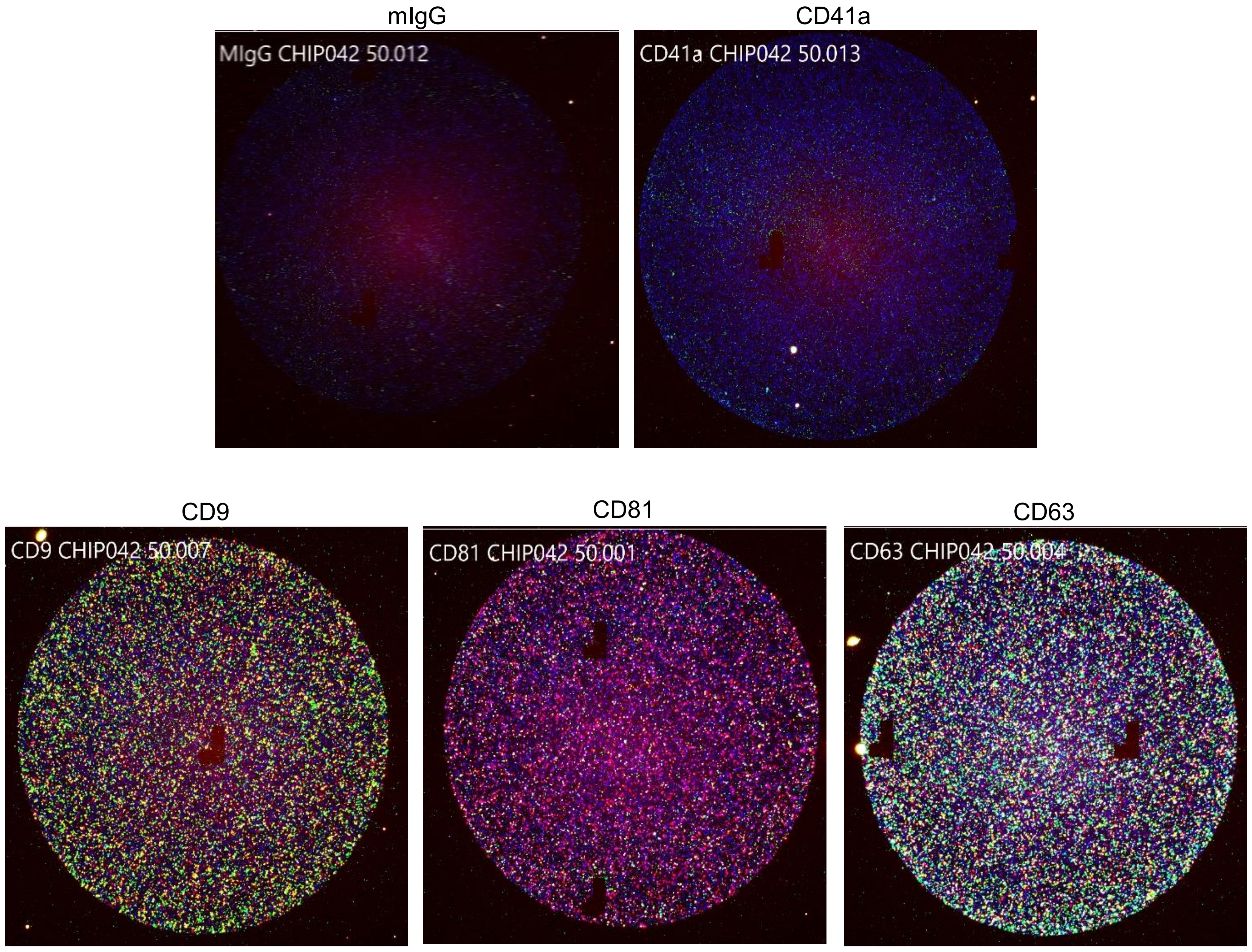
mIgG
MIgG CHIP042 50.012
CD41a
CD41a CHIP042 50.013
CD9
CD9 CHIP042 50.007
CD81
CD81 CHIP042 50.001
CD63
CD63 CHIP042 50.004

(b)

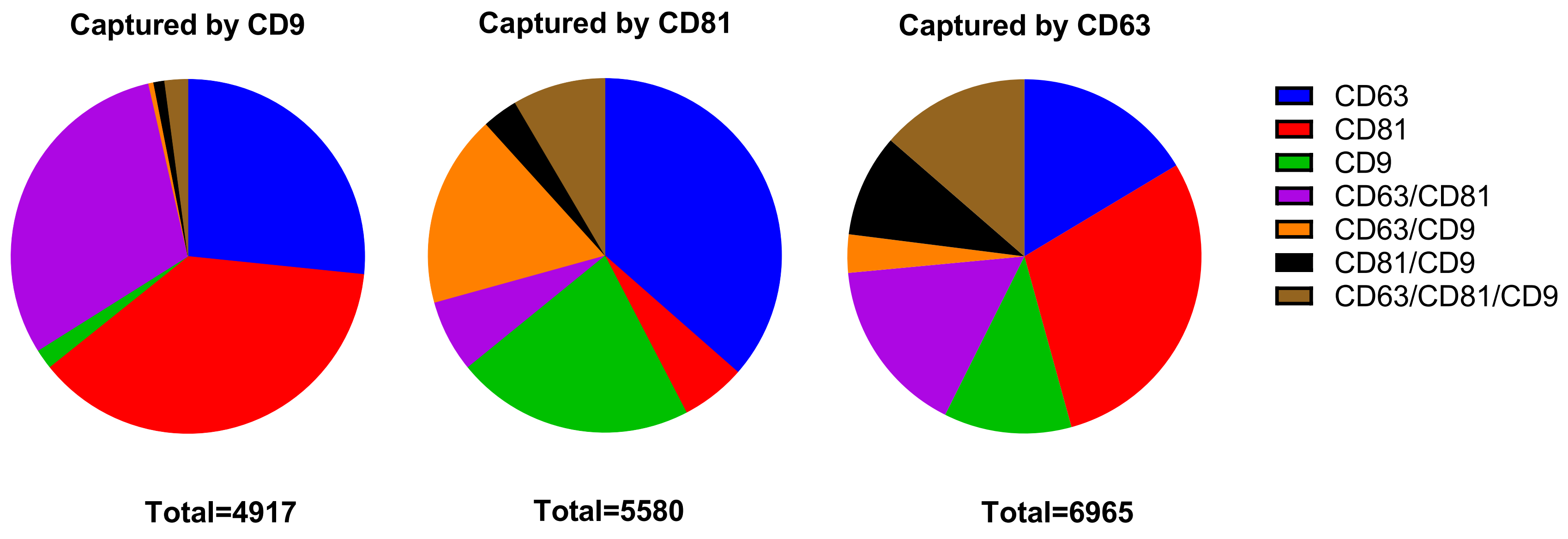
Captured by CD9
Captured by CD81
Captured by CD63
CD63
CD81
CD9
CD63/CD81
CD63/CD9
CD81/CD9
CD63/CD81/CD9
Total=4917
Total=5580
Total=6965
