# Supplementary material for: The Secretome of Human Trophoblast Stem Cells Attenuates Senescence‐Associated Traits
Source: Aging Cell. 2026 Jan 11;25(2):e70368. doi: 10.1111/acel.70368 (PMC12791570; doi:10.1111/acel.70368)
Supplement: Supplementary file 2 — Table S1: acel70368‐sup‐0002‐TableS1.zip. [file ACEL-25-e70368-s005.zip › Table S1.docx]

Table S1. RNA-sequencing analysis of differentially expressed transcripts. WI-38 fibroblasts were treated with ionizing radiation (IR, 10 Gy) and cultured for 5 days in hTSC-conditioned medium (hTSC-CM) or non-conditioned medium (NCM). RNA was then extracted and sequenced (GSE282054, Methods). The table indicates differentially abundant transcripts in hTSC-CM-treated cells compared to NCM-treated cells, reported as log2(fold change).
