# Supplementary material for: The Secretome of Human Trophoblast Stem Cells Attenuates Senescence‐Associated Traits
Source: Aging Cell. 2026 Jan 11;25(2):e70368. doi: 10.1111/acel.70368 (PMC12791570; doi:10.1111/acel.70368)
Supplement: Supplementary file 3 — Table S2: acel70368‐sup‐0003‐TableS2.zip. [file ACEL-25-e70368-s006.zip › Table S2.docx]

Table S2. Olink proteomic analysis of the WI-38 secretome. IR-treated WI-38 fibroblasts were cultured for 5 days in hTSC-CM or NCM. The secretomes of WI-38 cells were assessed by Olink proteomic analysis (Methods). The table indicates differentially abundant proteins in hTSC-CM-treated WI-38 cells compared to NCM-treated WI-38 cells, reported as normalized protein expression (NPX).
