# Supplementary material for: The Secretome of Human Trophoblast Stem Cells Attenuates Senescence‐Associated Traits
Source: Aging Cell. 2026 Jan 11;25(2):e70368. doi: 10.1111/acel.70368 (PMC12791570; doi:10.1111/acel.70368)
Supplement: Supplementary file 4 — Table S3: acel70368‐sup‐0004‐TableS3.zip. [file ACEL-25-e70368-s001.zip › Table S3.docx]

Table S3. Olink proteomic analysis of proteins enriched in hTSC-CM. Conditioned medium (CM) collected from cultured hTSCs and the corresponding non-conditioned medium (NCM) were assessed by Olink proteomic analysis (Methods). The table indicates proteins that are differentially abundant in hTSC-CM compared to NCM, reported as normalized protein expression (NPX).
