# Supplementary material for: The Secretome of Human Trophoblast Stem Cells Attenuates Senescence‐Associated Traits
Source: Aging Cell. 2026 Jan 11;25(2):e70368. doi: 10.1111/acel.70368 (PMC12791570; doi:10.1111/acel.70368)
Supplement: Supplementary file 5 — Table S4: acel70368‐sup‐0005‐TableS4.zip. [file ACEL-25-e70368-s003.zip › Table S4.docx]

Table S4. Mass spectrometry analysis to identify protein cargo in hTSC-derived EVs. Conditioned medium collected from cultured hTSCs was used for EV isolation and MS analysis. The table reports proteins detected in hTSC-EVs with values corresponding to normalized spectral abundance (MassIVE MSV000096320, Methods).
