# Supplementary material for: The Secretome of Human Trophoblast Stem Cells Attenuates Senescence‐Associated Traits
Source: Aging Cell. 2026 Jan 11;25(2):e70368. doi: 10.1111/acel.70368 (PMC12791570; doi:10.1111/acel.70368)
Supplement: Supplementary file 6 — Table S5: acel70368‐sup‐0006‐TableS5.zip. [file ACEL-25-e70368-s002.zip › Table S5.docx]

Table S5. Proteins enriched in hTSC-CM or hTSC-EVs that are downregulated in senescent cells. Integrated proteomic analysis identifying 148 proteins enriched in hTSC-CM or hTSC-derived EVs that were previously reported (by MS analysis) to be downregulated in senescent cells. The table shows the overlapping protein set and associated functional categories.
